# Single-cell RNA analysis to identify five cytokines signaling in immune-related genes for melanoma survival prognosis

**DOI:** 10.3389/fimmu.2023.1148130

**Published:** 2023-03-21

**Authors:** Zuhui Pu, Qing Zhao, Jiaqun Chen, Yubin Xie, Lisha Mou, Xushan Zha

**Affiliations:** ^1^ Imaging Department, Shenzhen Institute of Translational Medicine, The First Affiliated Hospital of Shenzhen University, Shenzhen Second People’s Hospital, Shenzhen, China; ^2^ Department of Dermatology, The First Affiliated Hospital of Guangzhou University of Chinese Medicine, Guangzhou, Guangdong, China; ^3^ Department of Dermatology, Shenzhen Luohu Hospital of Traditional Chinese Medicine, Shenzhen, Guangdong, China; ^4^ MetaLife Center, Shenzhen Institute of Translational Medicine, Shenzhen, Guangdong, China

**Keywords:** cytokine signaling in immune, prediction model, immune microenvironment, TMB, melanoma, GSEA, single-cell sequencing, machine learning

## Abstract

Melanoma is one of the deadliest skin cancers. Recently, developed single-cell sequencing has revealed fresh insights into melanoma. Cytokine signaling in the immune system is crucial for tumor development in melanoma. To evaluate melanoma patient diagnosis and treatment, the prediction value of cytokine signaling in immune-related genes (CSIRGs) is needed. In this study, the machine learning method of least absolute selection and shrinkage operator (LASSO) regression was used to establish a CSIRG prognostic signature of melanoma at the single-cell level. We discovered a 5-CSIRG signature that was substantially related to the overall survival of melanoma patients. We also constructed a nomogram that combined CSIRGs and clinical features. Overall survival of melanoma patients can be consistently predicted with good performance as well as accuracy by both the 5-CSIRG signature and nomograms. We compared the melanoma patients in the CSIRG high- and low-risk groups in terms of tumor mutation burden, infiltration of the immune system, and gene enrichment. High CSIRG-risk patients had a lower tumor mutational burden than low CSIRG-risk patients. The CSIRG high-risk patients had a higher infiltration of monocytes. Signaling pathways including oxidative phosphorylation, DNA replication, and aminoacyl tRNA biosynthesis were enriched in the high-risk group. For the first time, we constructed and validated a machine-learning model by single-cell RNA-sequencing datasets that have the potential to be a novel treatment target and might serve as a prognostic biomarker panel for melanoma. The 5-CSIRG signature may assist in predicting melanoma patient prognosis, biological characteristics, and appropriate therapy.

## 1 Introduction

Melanoma is the most dangerous type of skin cancer, accounting for 90% of all skin cancer deaths. Melanoma has become more common in recent decades, with an estimated 232,100 new cases and 55,500 deaths per year (1). Treatment of melanoma includes surgery, chemotherapy, radiotherapy, immunotherapy, targeted therapy, and other methods (2, 3). With a 90% cure rate, melanoma surgery remains the most effective treatment option. However, patients have a high rate of recurrence despite aggressive interventions, which contributes to the poor prognosis of melanoma (4). Therefore, new prognostic biomarkers for melanoma should be investigated to identify high-risk subpopulations and guide more effective individual treatments.

Lymphocytes, monocytes, macrophages, B cells, and T cells are immune system cells that create cytokines (5). Immune system cells can communicate with one another using cytokines to produce coordinated, efficient, but self-restraining antigen responses. Although there are numerous ways that the immune system can communicate with one another directly between cells, cytokine synthesis allows for a more varied and effective transmission of immunological information (6). Numerous malignancies, such as melanoma and renal cell carcinoma, are treated with cytokines (5). Cytokines at the tumor site stimulate immune effector cells, improving tumor cell recognition. The interaction between interleukins and interferons has an enhanced immunostimulatory impact (7). As a result, many cytokine- or cytokine antagonist-based cancer therapies have been developed (5, 8, 9). However, for patients with advanced-stage illness, cytokine-based therapy had a modest therapeutic effect.

Based on single-cell data and TCGA data, we used least absolute selection and shrinkage operator regression and Cox regression to build a prediction model of melanoma. The predictive significance of our cytokine signaling in immune-related genes (CSIRG) signature was further verified by receiver-operating characteristic analysis. Gene set enrichment analysis was conducted to help elucidate the intrinsic mechanisms. Moreover, the predictive significance of our CSIRG signature was confirmed using independent GEO data. Our results imply that the CSIRG signature plays a crucial role in predicting the prognosis of melanoma patients.

## 2 Methods

### 2.1 Data source

Melanoma single-cell datasets were accessed from GSE115978 (10). The TCGA (https://portal.gdc.cancer.gov/) (11) and GSE65904 cohorts (12), with 470 and 214 patients, respectively, provided bulk mRNA expression data in this study. Following data collection, an integrated analysis was conducted.

### 2.2 Data processing with single-cell RNA-sequencing

Seurat (version 4.1.0) was used to analyze the single-cell data of 31 melanoma patients. This cohort included 3 patients with primary melanoma and 28 patients with metastatic melanoma. Among these patients, 15 were untreated, and 16 were post-immunotherapy. The raw data were accessed from GSE115978 (10). Cells with > 600 and < 4,500 RNA features were included in the following analysis. Additionally, a linear dimensionality reduction was created using tSNE, and significant dimensions were identified with an estimated *P* value. Adjusted *P* < 0.05 and |logFC| > 0.25 were used for differentially expressed CSIRG analysis between metastatic melanoma and the primary melanoma group. We obtained cytokine signaling in immune-related genes (CSIRGs) from the Reactome pathway database.

### 2.3 Establishment of prognostic signature

The research included CSIRG signature genes with matching clinical characteristics. A training group (TCGA-SKCM) and a testing group (GSE65904) were formed from the melanoma patients. Univariate Cox regression was utilized in the TCGA-SKCM group to narrow down the prognostic CSIRG genes. The results from the univariate Cox regression were used for the least absolute selection and shrinkage operator (LASSO) regression. We further used the “glmnet” R package to perform the LASSO algorithm and select the potential candidates. A subsample of 1000 iterations was conducted on the dataset, and CSIRGs with occurrence frequencies of more than 950 were identified for further analysis. To narrow the list of candidate CSIRGs related to overall survival and construct a predictive gene signature, multivariate Cox regression was conducted. Finally, a risk score (RS) model of the CSIRG signature was constructed using the multivariate Cox results. Multivariate Cox regression was performed to acquire the regression coefficient (β), and the RS was generated using the coefficients and expressions. Using the findings, the RS equation is RS = coefficient1 * gene1 expression + coefficient2 * gene2 expression + coefficientN * geneN expression. Patients in the TCGA-SKCM and GSE65904 cohorts were divided into high- and low-risk groups by median RS. The survival analysis of melanoma patients was calculated using the Kaplan−Meier method. Receiver-operating characteristic (ROC) analyses validated the signature performance.

### 2.4 Survival analysis of 11 CSIRGs accessed from the results of LASSO regression

Kaplan–Meier survival analysis with the log-rank test was performed to study the 11 CSIRGs (*ATF2*, *CCR1*, *CRKL*, *EIF4A2*, *IFI30*, *MCL1*, *NUP188*, *STAT1*, *STAT3*, *TNFSF13B*, and *YWHAZ*) of melanoma patients in the TCGA-SKCM cohort. The patients were categorized into two high and low gene expression groups using the median expression level of each gene.

### 2.5 Nomogram construction

Before producing the nomogram, clinical and CSIRG signatures were combined. The appropriate clinical characteristics (including age, sex, tumor stage, tumor T stage, tumor M stage, and tumor N stage) and CSIRG RS were subsequently chosen using univariate and multivariate Cox regression models. The prognostic nomogram model was further developed using the CSIRG RS and independent clinical factors. ROC curves and decision curves were used to evaluate the performance of the nomogram model.

### 2.6 Mutation identification and tumor mutation burden quantification

TMB is base deletion, insertion, or substitution divided by the total number of variants divided by exon length. Maftools was used to analyze somatic mutation data to study TMB landscapes. The data were separated into two categories based on the CSIRG risk assessment. Mutant genes in the CSIRG high- and low-risk groups were analyzed.

### 2.7 Gene set enrichment analysis

Broad Institute’s software was used to conduct gene set enrichment analysis in the TCGA-SKCM cohort (13). A KEGG gene set (KEGG C2, MsigDB database v7.5.1) was used in this study. Gene set enrichment analysis was performed with 1,000 permutations (14). KEGG pathway study comparing the CSIRG high- and low-risk groups.

### 2.8 Immune infiltration analysis by xCell and CIBERSORT algorithm

We compared immune cell ratings using the xCell and CIBERSORT algorithms in the TCGA dataset (15, 16). To calculate the tumor-infiltrating cell distribution scores using xCell, this research integrated the transcriptome data of patients with the expression of marker genes from 64 different kinds of immune cells. Subgroup investigation of immune cell infiltration in the CSIRG high- and low-risk groups. To validate the xCell results, we further performed the most widely used immune infiltration algorithm of CIBERSORT to analyze the enrichment scores of 22 kinds of immune cells for each melanoma patient.

### 2.9 Statistical analysis

Statistical analyses and graphs were calculated using R version 4.0.5 and the necessary packages. *P* < 0.05 was considered to be statistically significant.

## 3 Results

### 3.1 Single-cell RNA sequencing analysis

**Figure 1:**
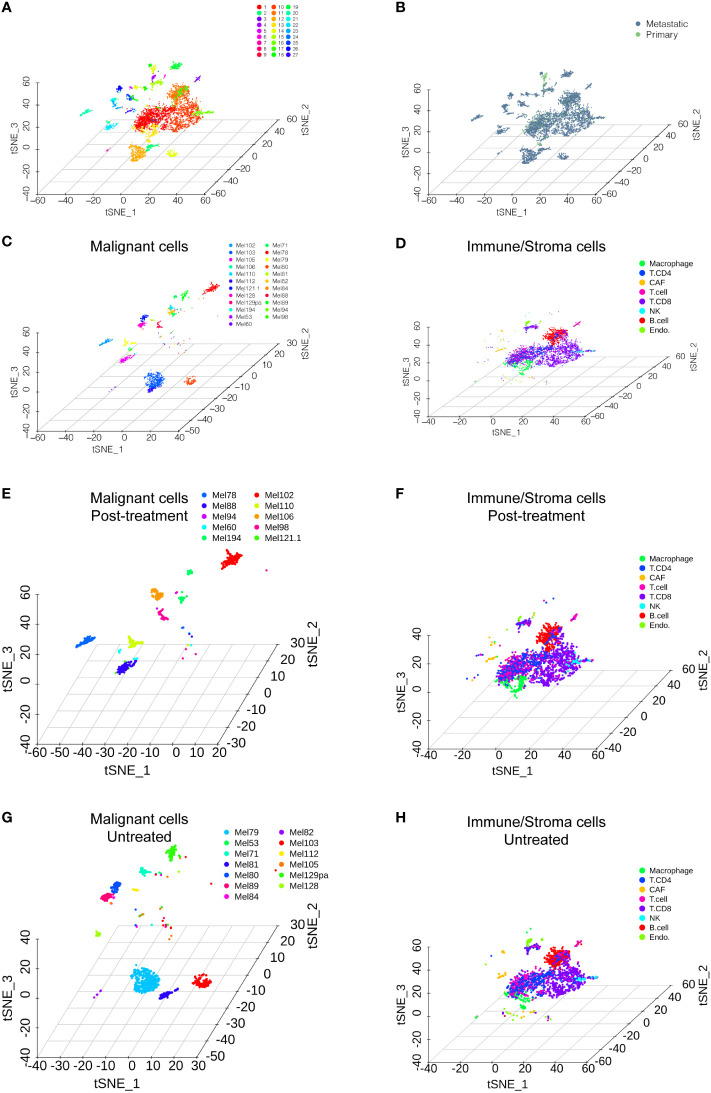
Single-cell RNA sequencing analysis of the GSE115978 dataset. **(A)** T-SNE of all of the melanoma samples with 27 clusters. **(B)** T-SNE of the metastatic melanoma and primary melanoma groups. **(C)** Malignant cells are categorized by the patients. **(D)** Nonmalignant cells, including immune cells and stromal cells, are classified by their cell type. The distribution of malignant cells and nonmalignant cells in the posttreatment group **(E, F)** and untreated group **(G, H)**.

We accessed the single-cell data of 31 patients from GSE115978 (10). Then, we performed single-cell transcriptome analysis to compare primary and metastatic melanoma by the Seurat pipeline. After quality control, a differential analysis was performed on the cells from the primary and metastatic melanoma tissues. The distribution of the single-cell data is shown in **Figures 1A–H**. The samples included 27 clusters (**Figure 1A**). We also showed the distribution of cells in different patient groups (**Figure 1B**, metastatic melanoma and primary melanoma groups). Malignant cells are categorized by patients (**Figure 1C**), while nonmalignant cells, including immune cells and stromal cells, are classified by their cell type (**Figure 1D**), as the original article reported. The nonmalignant cells included CD4+ T cells, CD8+ T cells, T cells, NK cells, macrophages cells, B cells, cancer-associated fibroblast (CAF) cells, and endothelial cells (**Figure 1D**). We further showed the distribution of malignant cells and nonmalignant cells in the posttreatment group (**Figures 1E–F**) and untreated group (**Figures 1G–H**). Among 31 patients, cells from only 23 patients were annotated with malignant cells which were shown in **Figures 1C, E, F**.

### 3.2 A risk model based on five cytokine signaling in immune-related genes

**Figure 2:**
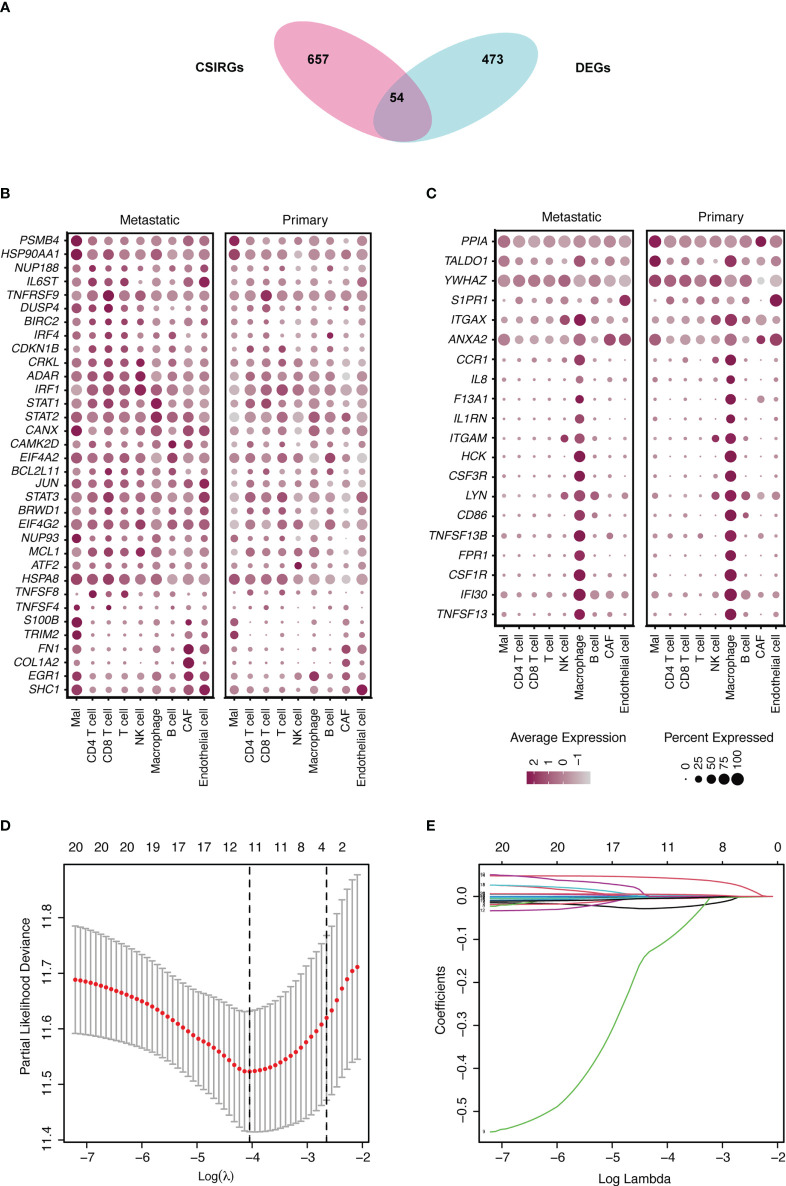
Identification of differentially expressed genes (DEGs) and cytokine signaling in immune-related genes (CSIRGs) between the metastatic melanoma and primary melanoma groups. **(A)** DEGs and CSIRGs in GSE115978 (|logFC| > 0.25 and adjusted *P* value < 0.05). **(B)** Dot plot showing the DEGs that were upregulated in the metastatic melanoma group. Mal: Malignant cells. **(C)** Dot plot showing the DEGs that were downregulated in the metastatic melanoma group. Mal: Malignant cells. **(D, E)** LASSO regression was performed on CSIRGs.

The CSIRGs used in the following analysis were obtained from the Reactome pathway database. As a consequence, 54 CSIRGs were differentially expressed between the metastatic melanoma and primary melanoma groups (**Figure 2A**). Compared with the primary melanoma group, 34 of the CSIRGs were upregulated in the metastatic melanoma group (**Figure 2B**; **Supplementary Table 1**), while 20 of the CSIRGs were downregulated in the metastatic melanoma group (**Figure 2C**; **Supplementary Table 1**). Among the upregulated CSIRGs, most were expressed in all of the cell types, including malignant cells (Mal), CD4+ T cells, CD8+ T cells, T cells, NK cells, macrophages cells, B cells, cancer-associated fibroblast (CAF) cells, and endothelial cells. However, *TNFSF8* was highly expressed in CD4+ T cells and T cells of both the primary and metastatic groups. *TNFSF4* was highly expressed in malignant cells and CD8+ T cells of the metastatic group. *S100B* and *TRIM2* were highly expressed in malignant cells and CAF cells of the metastatic group. *FN1* was highly expressed in Mal cells, CAF cells, and endothelial cells of the metastatic group. *COL1A2* was highly expressed in CAF cells of the metastatic group. *EGR1* was highly expressed in macrophage cells of the primary group. *SHC1* was highly expressed in endothelial cells of the primary group (**Figures 2B, C**).

**Figure 3:**
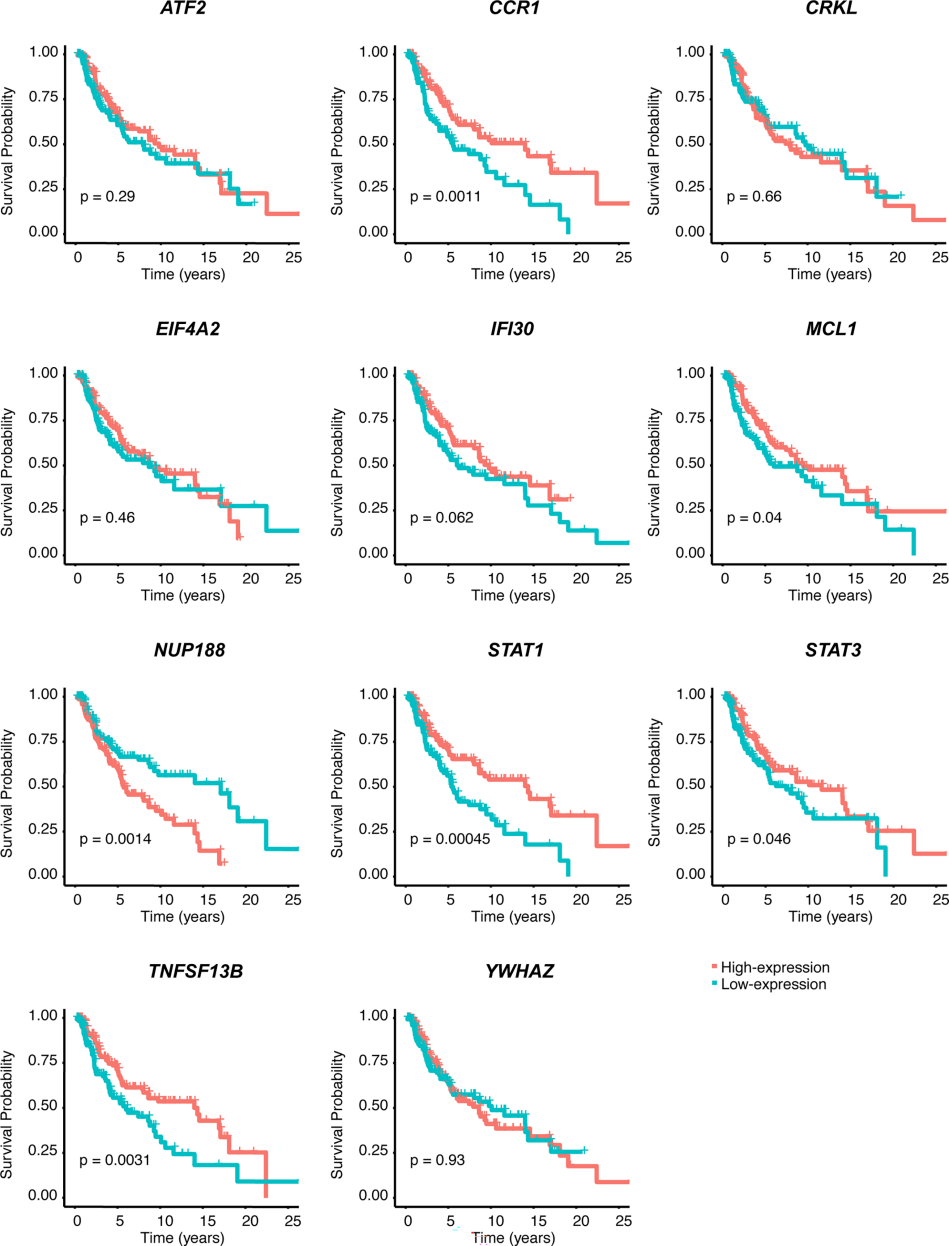
Kaplan–Meier survival analysis of 11 CSIRGs, including *ATF2, CCR1, CRKL, EIF4A2, IFI30, MCL1, NUP188, STAT1, STAT3, TNFSF13B, and YWHAZ*, identified by LASSO regression in the TCGA-SKCM cohort.

**Figure 4:**
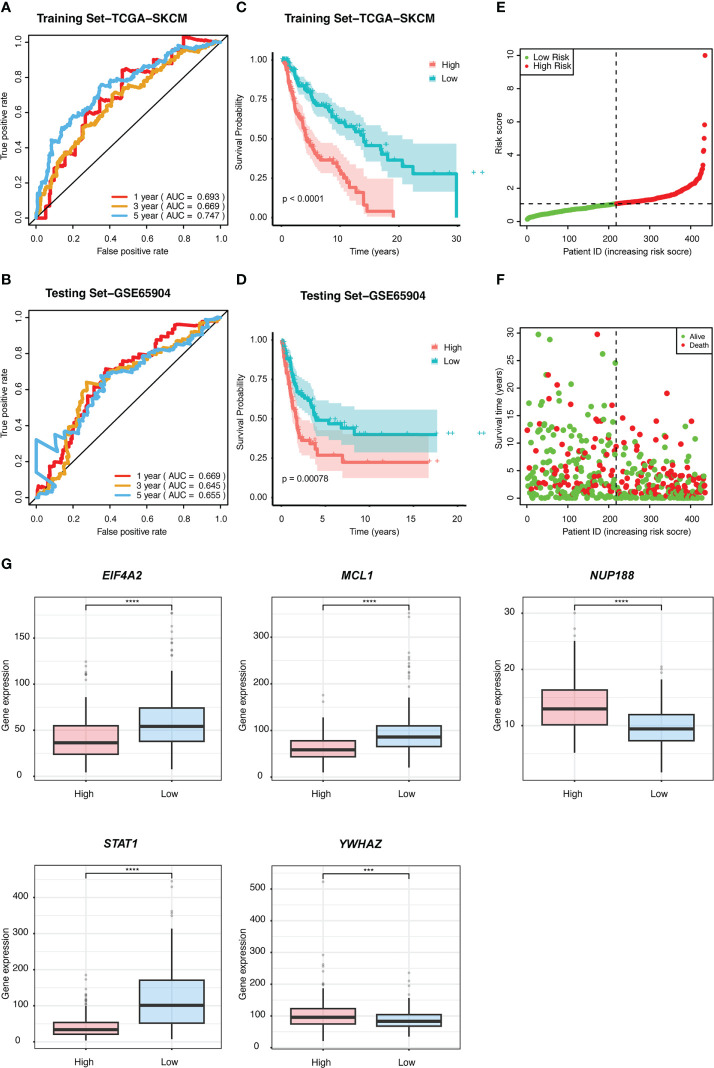
The prognostic value of the risk signature was validated. ROC curves for predicting 1-, 3-, and 5-year overall survival (OS) in the **(A)** TCGA and **(B)** GSE65904 datasets using the risk model. Kaplan−Meier curves were used to assess the OS probabilities in the training **(C)** and testing groups **(D)**. **(E)** In the training dataset of TCGA, the risk score (RS) of each patient is displayed. **(F)** OS and survival status are shown in the TCGA cohort (red dots indicate death, and green dots indicate survival). **(G)** The expression of the five CSIRGs (*EIF4A2*, *MCL1*, *NUP188*, *STAT1*, and *YWHAZ*) used to construct the prediction model was analyzed in the high- and low-risk groups. : ***: P≤0.001 and ****: P≤0.0001.

We performed univariate Cox regression analysis to screen prognostic CSIRGs from 54 CSIRGs. As a result, 20 CSIRGs were revealed to be highly linked with overall survival (OS) (**Supplementary Table 2**). LASSO regression further selected 11 CSIRGs (**Figures 2D, E**). Kaplan–Meier survival analysis of 11 CSIRGs, including *ATF2, CCR1, CRKL, EIF4A2, IFI30, MCL1, NUP188, STAT1, STAT3, TNFSF13B, and YWHAZ*, in melanoma patients in TCGA was performed. The results showed that high expression of *CCR1*, *MCL1*, *STAT1*, and *TNFSF13B* was correlated with longer survival time (**Figure 3**). High expression of *NUP188* was correlated with shorter survival time (**Figure 3**). Then, multivariate Cox regression analysis was carried out to screen prognostic CSIRGs from 11 CSIRGs. Finally, five hub CSIRGs (*EIF4A2*, *MCL1*, *NUP188*, *STAT1*, and *YWHAZ*) were identified with the minimum Akaike information criterion (AIC) and were further used for CSIRG model construction (**Table 1**; **Supplementary Table 3**). The gene model established based on five hub genes was as follows: risk score (RS) = -0.00536× *EIF4A2* - 0.00378 × *MCL1* + 0.05332 × *NUP188* - 0.00502 × *STAT1* + 0.00571 × *YWHAZ*. The RS medians (TCGA-SKCM: 1.071; GSE65904: 1.023) were utilized to divide the TCGA-SKCM and GSE65904 datasets into two groups with high and low RS (**Supplementary Tables 4**, **5**). The area under the receiver-operating characteristic curves (AUCs) proved that the gene model had relatively good accuracy. Specifically, the TCGA-SKCM dataset had AUCs of 0.693 (1 year), 0.669 (3 years), and 0.747 (5 years) (**Figure 4A**), whereas the GSE65904 dataset had AUCs of 0.669 (1 year), 0.645 (3 years), and 0.655 (5 years) (**Figure 4B**). Furthermore, Kaplan−Meier curves of both the TCGA-SKCM (**Figure 4C**) and GSE65904 datasets (**Figure 4D**) revealed that melanoma patients in the CSIRG high-risk group were associated with worse outcomes. The RS of each patient in the TCGA-SKCM cohort is displayed in **Figure 4E**. **Figure 4F** also confirmed that the mortality of the CSIRG high-risk melanoma patients increased. The expression of the five hub CSIRGs (*EIF4A2*, *MCL1*, *NUP188*, *STAT1*, and *YWHAZ*) in the high- and low-risk groups is shown in **Figure 4G**. The results showed that *EIF4A2*, *MCL1*, and *STAT1* were downregulated in the high-risk group, while *NUP188* and *YWHAZ* were upregulated in the high-risk group (**Figure 4G**).

**Table 1:** Multivariate cox regression analysis of different combination of CSIRGs by the Akaike information criterion (AIC).

Combination	AIC
*ATF2, CCR1, CRKL, EIF4A2, IFI30, MCL1, NUP188, STAT1, STAT3, TNFSF13B, YWHAZ*	1483.79
*ATF2, CRKL, EIF4A2, IFI30, MCL1, NUP188, STAT1, STAT3, TNFSF13B, YWHAZ*	1481.89
*ATF2, CRKL, EIF4A2, IFI30, MCL1, NUP188, STAT1, TNFSF13B, YWHAZ*	1480.23
*ATF2, CRKL, EIF4A2, IFI30, MCL1, NUP188, STAT1, YWHAZ*	1478.98
*ATF2, EIF4A2, IFI30, MCL1, NUP188, STAT1, YWHAZ*	1478.27
*ATF2, EIF4A2, MCL1, NUP188, STAT1, YWHAZ*	1477.84
*EIF4A2, MCL1, NUP188, STAT1, YWHAZ*	1477.38

### 3.3 Nomogram model construction for melanoma patients

**Figure 5:**
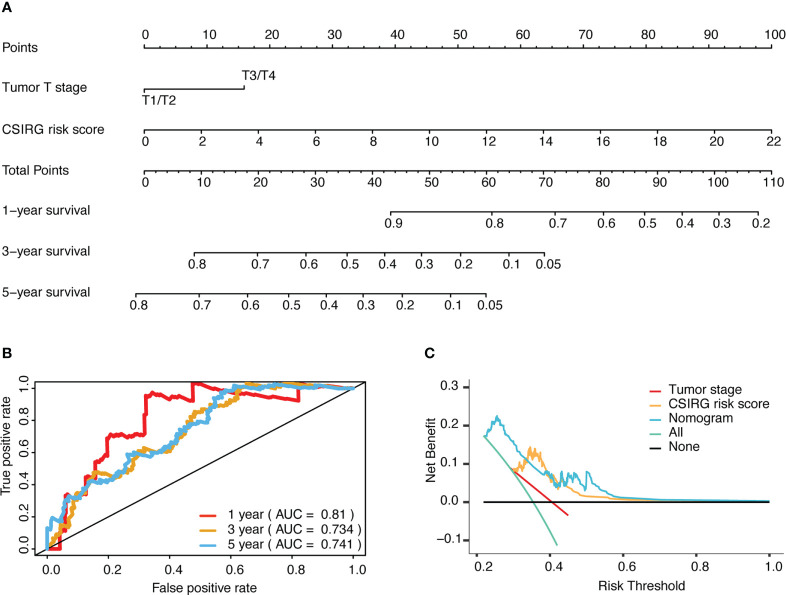
Development and evaluation of a risk-based predictive nomogram for melanoma. **(A)** A nomogram for predicting melanoma prognosis that includes the CSIRG RS and tumor T stage. **(B)** Receiver operating characteristic curves and the area under the receiver operating characteristic curve evaluation of the nomogram performance of 1-, 3-, and 5-year OS. **(C)** Decision curve evaluation of the nomogram performance for 5-year OS.

To further construct a nomogram combining clinical variables (including age, sex, tumor stage, tumor T stage, tumor M stage, and tumor N stage) and CSIRG RS, univariate and multivariate Cox regression models were constructed from the TCGA-SKCM dataset (**Table 2**). As a result, the tumor T stage was selected and integrated with the RS to create a clinical nomogram using the generalized linear model regression technique (**Figure 5A**). The survival rates of melanoma patients over 1, 3, and 5 years could be predicted using the clinical nomogram. The AUCs for 1, 3, and 5 years were 0.81, 0.734, and 0.741 for the TCGA-SKCM dataset, respectively (**Figure 5B**). Decision curve analysis was carried out to evaluate the nomogram performance for 5-year OS (**Figure 5C**).

**Table 2:** Univariate and multivariate Cox regression to analysis independent prognosis factors in the TCGA-SKCM cohort.

Characteristics	Univariate Cox			Multivariate Cox		
	HR (95% CI)	* P* value	FDR	HR (95% CI)	* P* value	FDR
Age (≥ 60 *vs.*<60)	1.57 (1.07-2.29)	**2.02E-02**	** 2.83E-02**	1.37 (0.92-2.04)	1.20E-01	2.09E-01
Gender (Male *vs.* Female)	0.97 (0.65-1.43)	8.64E-01	8.64E-01	1.04 (0.69-1.56)	8.55E-01	8.55E-01
Tumor stage (III/IV *vs.* I/II)	1.78 (1.21-2.61)	**3.49E-03**	**8.15E-03**	1.6 0(1.00-2.56)	5.09E-02	1.19E-01
Tumor T stage (T3/T4 *vs.* T1/T2)	2.09 (1.42-3.08)	**1.94E-04**	**6.80E-04**	1.81 (1.20-2.73)	**4.81E-03**	**1.68E-02**
Tumor M stage (M1 *vs.* M0)	2.06 (0.75-5.61)	1.59E-01	1.86E-01	1.74 (0.61-4.91)	2.99E-01	4.07E-01
Tumor N stage (N2/N3 *vs.* N0/N1)	1.76 (1.10-2.80)	**1.79E-02**	**2.83E-02**	1.32 (0.74-2.34)	3.49E-01	4.07E-01
CSIRG risk score (High *vs.* Low)	1.23 (1.14-1.32)	**2.61E-08**	**1.83E-07**	.25 (1.16-1.35)	**2.08E-08**	**1.46E-07**

P<0.05 were shown in the bold values.

### 3.4 Tumor mutation burden analysis

**Figure 6:**
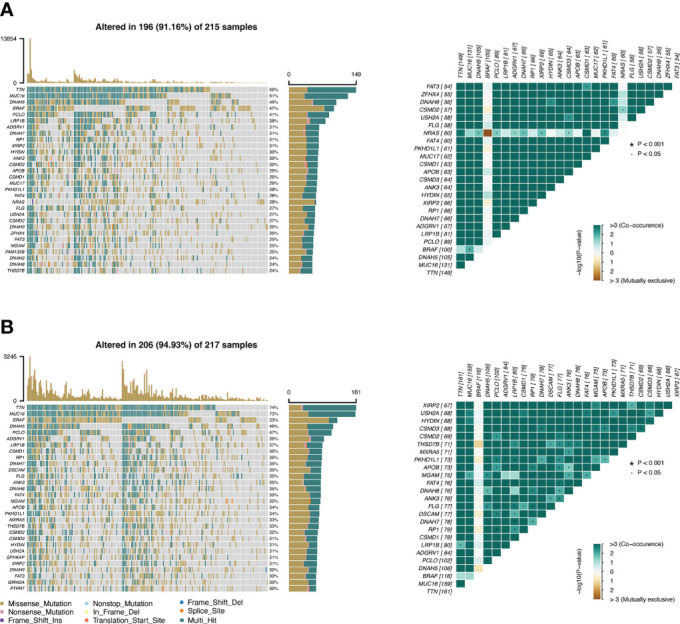
The difference in tumor mutation burden between the CSIRG low- and high-score groups is shown. **(A, B)** Gene mutation information in the high-risk **(A)** and low-risk **(B)** categories of the TCGA dataset.

The TMB profile of the TCGA-SKCM dataset was downloaded and matched with the RS for subsequent analysis. *TTN*, *MUC16*, *DNAH5*, *BRAF*, and *PLCO* were the top five mutated genes in the CSIRG high- and low-risk groups, and their mutation rates were higher in the low-risk group than in the high-risk group (**Figures 6A, B**). The sixth mutated gene in the CSIRG high-risk group was *LRP1B*, and the mutation rate of this gene was higher in the high-risk group (38%) than in the low-risk group (39%) (**Figures 6A, B**). Since *TTN* demonstrated the highest mutation (high risk: 69%, low risk: 74%) in the TCGA-SKCM dataset, it might be an important risk factor in melanoma (**Figures 6A, B**).

### 3.5 Gene set enrichment analysis

**Figure 7:**
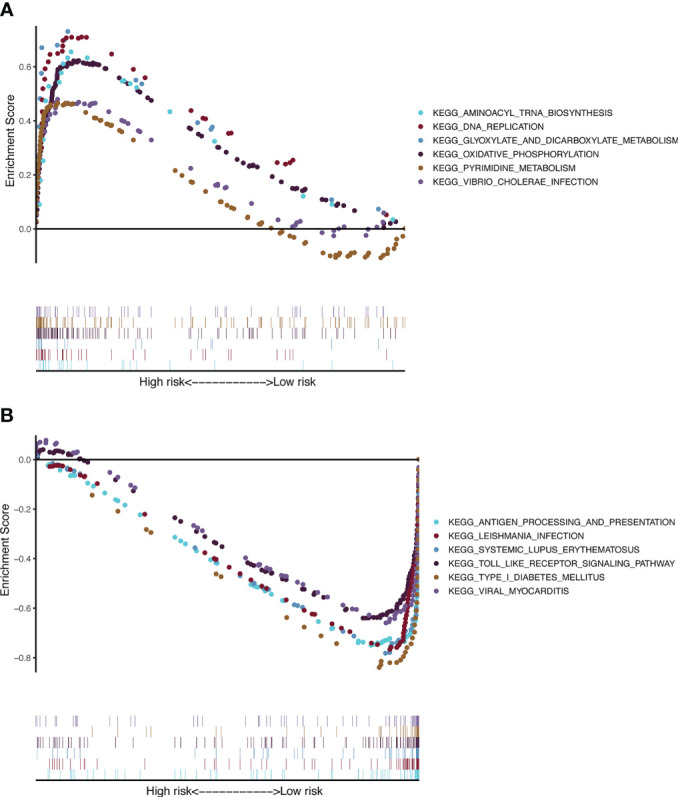
KEGG and hallmark gene set enrichment analysis for the CSIRG high-risk **(A)** and low-risk **(B)** melanoma patient groups in the TCGA cohort.

To gain further insight into signaling pathways associated with melanoma, GSEA was performed on the TCGA-SKCM dataset (**Supplementary Table 6**). The results from GSEA revealed that aminoacyl tRNA biosynthesis, DNA replication, glyoxylate and dicarboxylate metabolism, oxidative phosphorylation, pyrimidine metabolism, and Vibrio cholerae infection were mainly enriched in the high-risk group (**Figures 7A**). In contrast, antigen processing and presentation, Leishmania infection, systemic lupus erythematosus, Toll-like receptor signaling pathway, type 1 diabetes mellitus, and viral myocarditis were mainly enriched in the low-risk group (**Figures 7B**).

### 3.6 Immune cell infiltration analysis

**Figure 8:**
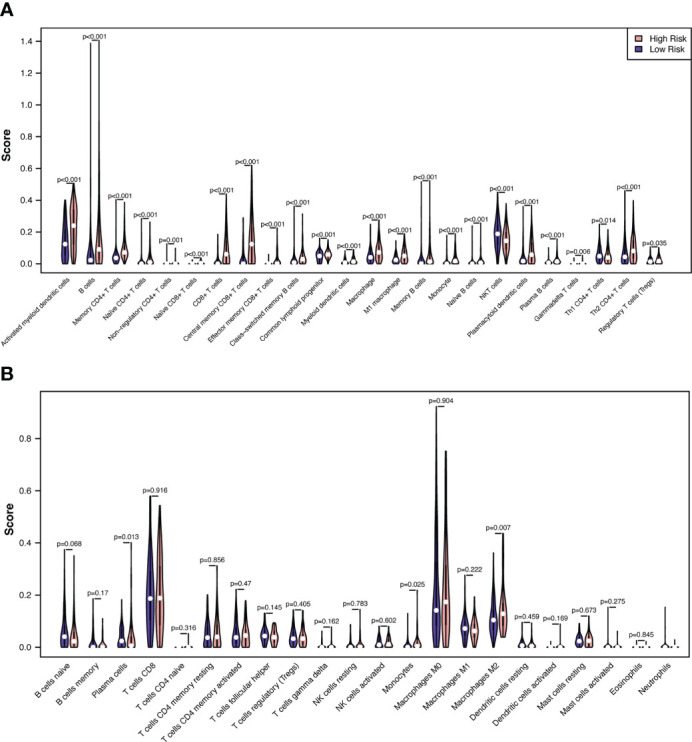
Correlations between melanoma RS and tumor immune microenvironment. The proportionate differences in immune and stromal cells between the CSIRG high- and low-risk melanoma patient groups were visualized using a violin plot by the xCell **(A)** and CIBERSORT **(B)** algorithms.

We then evaluated the influence of the RS on the immune microenvironment by comparing immune cell infiltration in the TCGA-SKCM dataset through the xCell algorithm. Increased enrichment of activated myeloid dendritic cells, B cells, memory CD4+ T cells, naïve CD4+ T cells, nonregulatory CD4+ T cells, naïve CD8+ T cells, CD8+ T cells, central memory CD8+ T cells, effector memory CD8+ T cells, class-switched memory B cells, common lymphoid progenitors, myeloid dendritic cells, macrophages, M1 macrophages, memory B cells, monocytes, naïve B cells, plasmacytoid dendritic cells, plasma B cells, gamma-delta T cells, Th2 CD4+ T cells, and regulatory T cells was found in the high-risk group, while increased enrichment of NKT cells and Th1 CD4+ T cells was found in the low-risk group (**Figure 8A**; **Supplementary Table 7**).

To validate the results of xCell, we further used the CIBERSORT algorithm to analyze immune cell infiltration. The results showed that increased enrichment of monocytes and M2 macrophages was found in the high-risk group, while increased enrichment of plasma cells was found in the low-risk group (**Figure 8B**). As a result, only monocytes were found to be positively correlated with the high-risk group in both the xCell and CIBERSORT algorithms.

## 4 Discussion

Previous research has shown that the prognoses of melanoma patients cannot be adequately predicted using routinely utilized clinicopathological markers. In this study, five cytokine signaling pathways in immune-related genes (CSIRGs, including *EIF4A2, MCL1, NUP188, STAT1*, and *YWHAZ*) were used to generate a predictive signature. This signature was used to classify the melanoma patients in the TCGA and GSE65904 cohorts into low-risk and high-risk groups, depending on their risk scores. In addition, the results of the receiver-operating characteristic (ROC) analysis demonstrated that the five-gene signature has good potential for predicting overall survival in melanoma patients. The expression of *EIF4A2* has also been shown to have a favorable correlation with the prognosis of non-small cell lung cancer and breast cancer in a number of studies (17, 18). On the other hand, a recent study found that higher *EIF4A2* expression in colorectal cancer (CRC) was linked to a worse chance of survival. In addition, the findings of research conducted on cells and animals have provided additional evidence that *EIF4A2* has a role in both the promotion of CRC metastasis and oxaliplatin resistance (19). An antiapoptotic protein that promotes resistance to numerous chemotherapeutic agents (20) is encoded by the *MCL1* gene, which is typically increased in melanoma as well as in many other kinds of tumors. Nucleoporin is a component of the nuclear pore complex, which is encoded by the *NUP188* gene (NPC). In mitotic cells, nucleoporin plays a role in the regulation of chromosomal segregation by increasing chromosome alignment. It is possible that problems with the chromosomal segregation process are responsible for the aneuploidy that occurs in some cancer cells (21). Therefore, *NUP188* might play a part in the process of oncogenesis. Studies have also demonstrated that the expression of *STAT1* is lower in gliomas than in normal brain tissues (22, 23). This difference in expression has been proven to occur in gliomas. The overexpression of *STAT1* significantly inhibits the development of glioma cells and stimulates apoptosis (24) in these cells. However, the function of aberrant *STAT1* and the mechanism by which it causes melanoma are not yet fully understood. Emerging data have shown that *YWHAZ* is essential in the growth of many different kinds of tumors (25). *YWHAZ* has also been shown to be a useful prognostic marker for multiple cancers according to a number of studies (26–29).

We further analyzed the correlation of tumor mutation burden (TMB) in the CSIRG high- and low-risk groups. Patients in the high-TMB group had significantly better survival outcomes. The mutation of *TTN*, which can aid carcinogenesis and metastasis in melanoma, was proven to be the most common mutation in melanoma patients. We showed that *TTN* mutation might lead to a good overall outcome for melanoma patients in the low-risk group. The mutation of TTN, which should be investigated in further studies, was negatively correlated with RS in our study.

The association of immune cell infiltration with risk score was explored, and the modulation of immune cells by CSIRGs is relatively underexplored in skin melanoma. We compared the immunological microenvironments (TMEs) of the CSIRG high- and low-risk groups and discovered that the former was enriched with monocytes. Although monocytes were more enriched in the high-risk group, survival was worse in the high-risk group. Previous studies proved that there are functionally distinct subsets of monocytes in different tumor microenvironments (30, 31). Pathologically activated monocytes are myeloid-derived suppressor cells (MDSCs) that have potent immunosuppressive effects (32). Important differences between MDSCs and classical monocytes were also reported (32, 33). In addition to causing tumor vasculogenesis, MDSCs suppress T-cell immunity and enable tumors to escape immunity (33). (PMID: 24060865). A previous study also proved that a poor prognosis was associated with phosphorylated STAT3 expression in monocytes (34). Aberrantly hyperactivated STAT3 monocytes promote liver tumorigenesis in both clinical patients and *in vivo* animal experiments (34). It is possible that the enriched monocytes in the CSIRG high-risk group have the same characteristics as MDSCs, which have potent immunosuppressive activity.

CSIRG may participate in the TME and immunological responses. Among the five CSIRGs, *STAT1* and *MCL1* were previously reported to be associated with infiltrating immune cells. JAK1/STAT1 activation occurs in immune-exhausted cells and is associated with increased Treg cell scores and upregulation of PDL1 (35). *STAT1 in* the majority of immune cell populations was more active in the high-risk group, but survival was worse. It was also reported that *STAT1*-deficient mice exhibited decreased accumulation of Th1 cells (36). Moreover, *STAT1* is a biomarker of immune infiltration changes after anti-tuberculosis treatment (37). *STAT1* was positively correlated with monocytes and neutrophils and negatively correlated with CD8+ T cells (37). In a study of acute lung injury, *MCL1* was found to be negatively correlated with both B cells and T cells (38). Further in-depth research is required to study the relationship between CSIRG and the TME.

We further applied GSEA to detect the genet set enrichment in the CSIRG high- and low-risk groups. KEGG pathway analysis showed that these genes are associated with cytokine signaling in immune pathways. Enrichment with phenotypic consistency was also found in pathways such as glyoxylate and dicarboxylate metabolism, oxidative phosphorylation, DNA replication, aminoacyl tRNA biosynthesis, and pyrimidine metabolism, which were enriched in the high-risk group, whereas antigen processing and presentation, the Toll-like receptor signaling pathway and viral myocarditis were enriched in the low-risk group. Above all, it is reasonable to suggest that these CSIRGs might participate in the occurrence and development of melanoma through these pathways.

In summary, we defined a novel CSIRG signature in melanoma. This CSIRG signature and nomogram showed high predictive capability for the prognosis of melanoma patients. New insights into the prognosis of patients with melanoma may be provided by the CSIRG signature. Melanoma therapeutic targets may be developed through pathways related to CSIRG. Patients may benefit from further screening of anticancer drugs sensitive to both high and low CSIRG groups.

## Data availability statement

The original contributions presented in the study are included in the article/**Supplementary Material**. Further inquiries can be directed to the corresponding authors.

## Author contributions

ZP and LM initiated the study. QZ and LM performed the data analysis and manuscript writing. JC and YX revised the manuscript. XZ supervised the study. All authors contributed to the article and approved the submitted version.
